# Cardiac magnetic resonance for asymptomatic patients with type 2 diabetes and cardiovascular high risk (CATCH): a pilot study

**DOI:** 10.1186/s12933-020-01019-2

**Published:** 2020-03-31

**Authors:** Ming-Yen Ng, Wenli Zhou, Varut Vardhanabhuti, Chi-Ho Lee, Esther Yee Tak Yu, Eric Yuk Fai Wan, Kit Chan, Andrew T. Yan, Tai-Pang Ip, Kai-Hang Yiu, Bernd J. Wintersperger

**Affiliations:** 1 Department of Diagnostic Radiology, The University of Hong Kong, Room 406, Block K, 102 Pokfulam Road, Hong Kong SAR, China; 2 Department of Medical Imaging, The University of Hong Kong-Shenzhen Hospital, Shenzhen, China; 3 Department of Medicine, The University of Hong Kong, Hong Kong, China; 4 Department of Family Medicine and Primary Care, The University of Hong Kong, Hong Kong, China; 5 Department of Cardiology, The University of Hong Kong-Shenzhen Hospital, Shenzhen, China; 6 Departments of Medicine and Medical Imaging, St. Michael’s Hospital, Toronto, Canada; 7 University of Toronto, Toronto, Canada; 8 Department of Medicine, Tung Wah Hospital, Hong Kong, China; 9 Department of Medical Imaging, University of Toronto, Toronto, Canada; 10 Department of Medical Imaging, University Health Network, Toronto, Canada

**Keywords:** Stress cardiac magnetic resonance, Type 2 diabetes, Asymptomatic, High cardiovascular risk, Screening

## Abstract

**Background:**

Stress cardiovascular magnetic resonance (CMR) to screen for silent myocardial ischaemia in asymptomatic high risk patients with type 2 diabetes mellitus (DM) has never been performed, and its effectiveness is unknown. Our aim was to determine the feasibility of a screening programme using stress CMR by obtaining preliminary data on the prevalence of silent ischaemia caused by obstructive coronary artery disease (CAD) and quantify myocardial perfusion in asymptomatic high risk patients with type 2 diabetes.

**Methods:**

In this prospective cohort study, we recruited 63 asymptomatic DM patients (mean age 66 years ± 4.4 years; 77.8% male); with Framingham risk score ≥ 20% from 3 sites from June 2017 to August 2018. Normal volunteers were recruited to determine normal global myocardial perfusion reserve index (MPRI). Adenosine stress CMR and global MPRI was performed and measured in all subjects. Positive stress CMR cases were referred for catheter coronary angiography (CCA) with/without fractional flow reserve (FFR) measurements. Positive CCA was defined as an FFR ≤ 0.8 or coronary narrowing ≥ 70%. Patients were followed up for major adverse cardiovascular events. Prevalence is presented as patient numbers and percentage. Mann–Whitney U test was used to compare global MPRI between patients and normal volunteers.

**Results:**

13 patients had positive stress CMR with positive CCA (20.6% of patient population), while 9 patients with positive stress CMR examinations had a negative CCA. 5 patients (7.9%) had infarcts detected of which 2 patients had no stress perfusion defects. 12 patients had coronary artery stents inserted, whilst 1 patient declined stent placement. DM patients had lower global MPRI than normal volunteers (n = 7) (1.43 ± 0.27 vs 1.83 ± 0.31 respectively; p < 0.01). After a median follow-up of 653 days, there was no death, heart failure, acute coronary syndrome hospitalisation or stroke.

**Conclusion:**

20.6% of asymptomatic DM patients (with Framingham risk ≥ 20%) had silent obstructive CAD. Furthermore, asymptomatic patients have reduced global MPRI than normal volunteers.

*Trial Registration:* ClinicalTrials.gov Registration Number: NCT03263728 on 28th August 2017; https://clinicaltrials.gov/ct2/show/NCT03263728.

## Background

Asymptomatic coronary artery disease (CAD) is highly prevalent (i.e. 17–59%) in patients with diabetes mellitus type 2 (DM) [1]. In addition, cardiovascular disease remains the most common cause of death in DM patients [2]. Previous trials using coronary computed tomography angiograms or nuclear myocardial perfusion imaging to screen for asymptomatic obstructive CAD requiring intervention have been unsuccessful at reducing cardiovascular and all-cause mortality, when compared to optimised medical therapy where cardiovascular risk factors are treated to reduce cardiovascular complications [3, 4]. Possible reasons for this include, the choice of imaging modality, the intervention chosen (e.g. bare metal stents vs drug eluting stents), anatomical or fractional flow reserve (FFR) guidance and patient cohort (e.g. unselected DM patients vs high risk DM patients). Stress cardiac magnetic resonance (CMR) is ideally suited to assess this group of high risk patients, as there is no radiation exposure and it allows a more complete cardiac assessment including myocardial viability, left ventricular systolic and diastolic function. It has also shown to be non-inferior to FFR [5]. Nevertheless, a study using stress CMR to screen for myocardial ischaemia with catheter coronary angiography confirmation followed by intervention has never been performed. Thus the effectiveness of stress CMR to identify asymptomatic DM patients with silent ischaemia and its impact on patient outcomes are unknown.

Stress perfusion CMR identifies hemodynamically relevant coronary artery disease (CAD) on the basis of stress induced perfusion defects in the downstream supply territory. Furthermore, stress perfusion CMR enables the quantification of myocardial perfusion allowing for detection of underlying microvascular disease in the absence of obstructive CAD [6]. Previous studies quantifying myocardial perfusion have indicated that myocardial perfusion quantification is a useful prognostic marker of patient outcome [7]. Moreover, in DM patients, the presence of microvascular disease (MCAD) without obstructive CAD carries similar risk of adverse events as non-diabetic patients with obstructive CAD [7]. Global myocardial perfusion reserve index (MPRI) is a semi-quantifiable parameter which can be determined on stress perfusion CMR and has been shown to be diagnostic of MCAD [6]. Global MPRI provides information of the overall myocardial perfusion but does not differentiate epicardial CAD or MCAD. Stress-induced perfusion defects identified visually are usually suggestive of epicardial CAD whilst its absence is indicative of no significant epicardial CAD. However, when the search of stress induced perfusion defects visually is combined with global MPRI, additional diagnostic information can be provided by inferring the presence of MCAD when global MPRI is reduced and there is an absence of stress induced perfusion defects [6].

In order to determine the feasibility of a larger randomised controlled trial design for assessing the effectiveness of stress perfusion CMR, our study has two aims: (1) to determine the prevalence of myocardial ischaemia confirmed with catheter coronary angiography in asymptomatic high risk DM patients using stress CMR screening; (2) to quantify myocardial perfusion (i.e. MPRI) in asymptomatic high risk DM patients compared to healthy volunteers.

## Materials and methods

The Cardiac Magnetic Resonance for Asymptomatic Patients with Type 2 Diabetes with Cardiovascular High Risk (CATCH) study (clinicalTrials.org: NCT03263728) was designed as a prospective cohort study. The study was approved by the local research ethics committee.

Patients were recruited consecutively from June 2017 to August 2018 at two diabetes clinics (n = 58) and one family medicine clinic (n = 5). All patients gave informed consent to be enrolled in the study.

Inclusion criteria were aged 60–80 years old, onset of DM at ≥ 30years old with no history of ketoacidosis, and Framingham Risk Score ≥ 20% (i.e. High risk based on Framingham Risk Score) [8]. Exclusion criteria were angina pectoris or chest discomfort, stress test or coronary angiography within 2 years, previous myocardial infarction, previous coronary artery stenting or bypass grafting, any clinical indication or contraindication for stress testing, any contraindication to stress CMR (e.g. previous anaphylaxis to adenosine), contraindication to gadolinium based contrast agent (e.g. Renal impairment with an estimated glomerular filtration rate < 30 ml/min/1.73 m^2^, life expectancy < 2 years due to cancer or liver disease, contraindication to dual antiplatelet therapy, planned concomitant cardiac surgery, refusal or inability to provide informed consent and potential for non-compliance of the trial protocol.

**Fig. 1:**
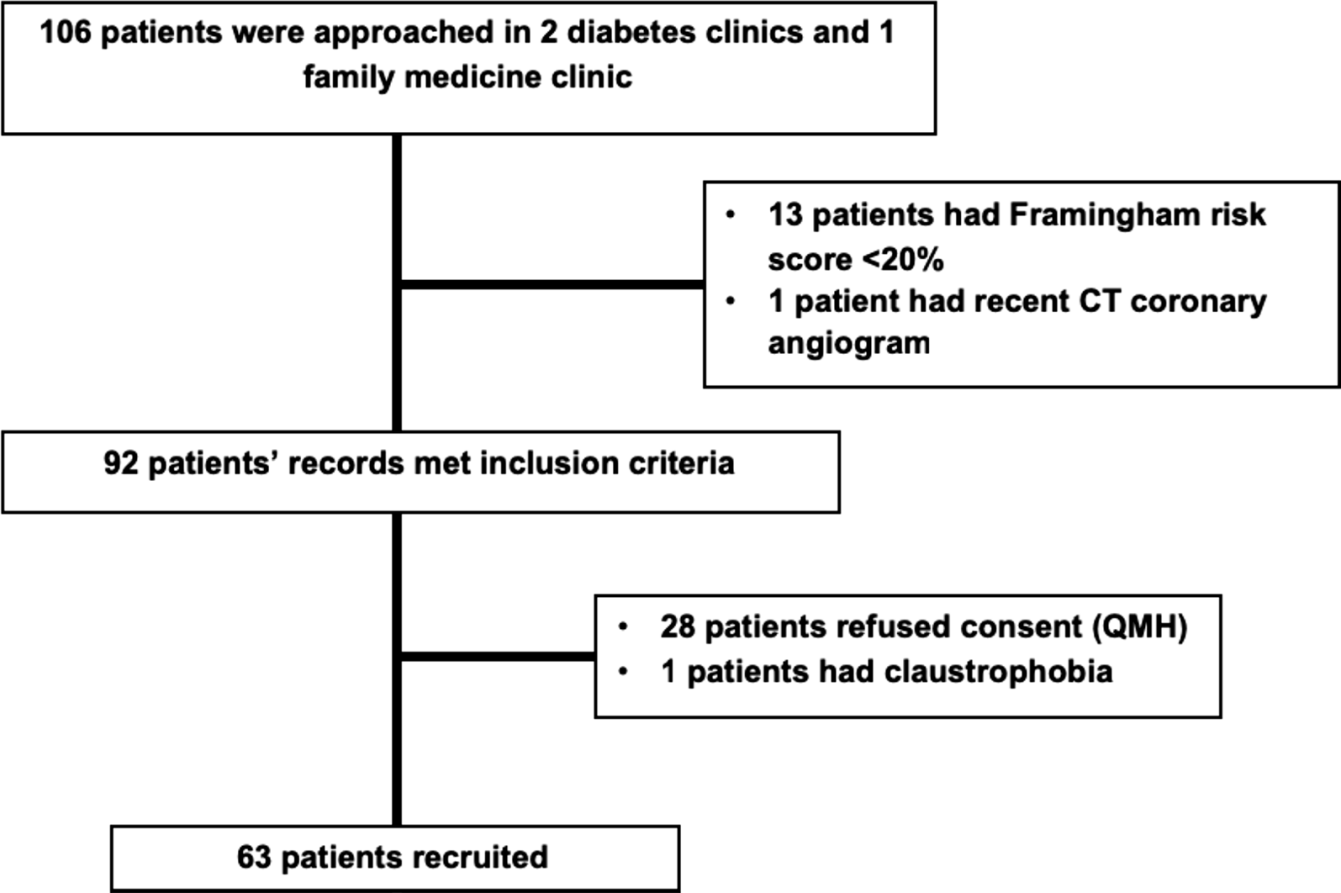
CONSORT diagram

63 patients (mean age 66 years, range 60–78 years; 77.8% male) were enrolled into the study (see Fig. 1). 7 healthy volunteers aged ≥ 18 years were recruited. Volunteers were deemed healthy if they had no cardiac symptoms or risk factors, no known cardiac disease, normal electrocardiogram, systolic and diastolic blood pressure < 140 mmHg and < 100 mmHg respectively, normal fasting glucose, normal brain-natriuretic peptide and normal stress CMR examination.

All study participants provided written informed consent.

### Workflow

**Fig. 2:**
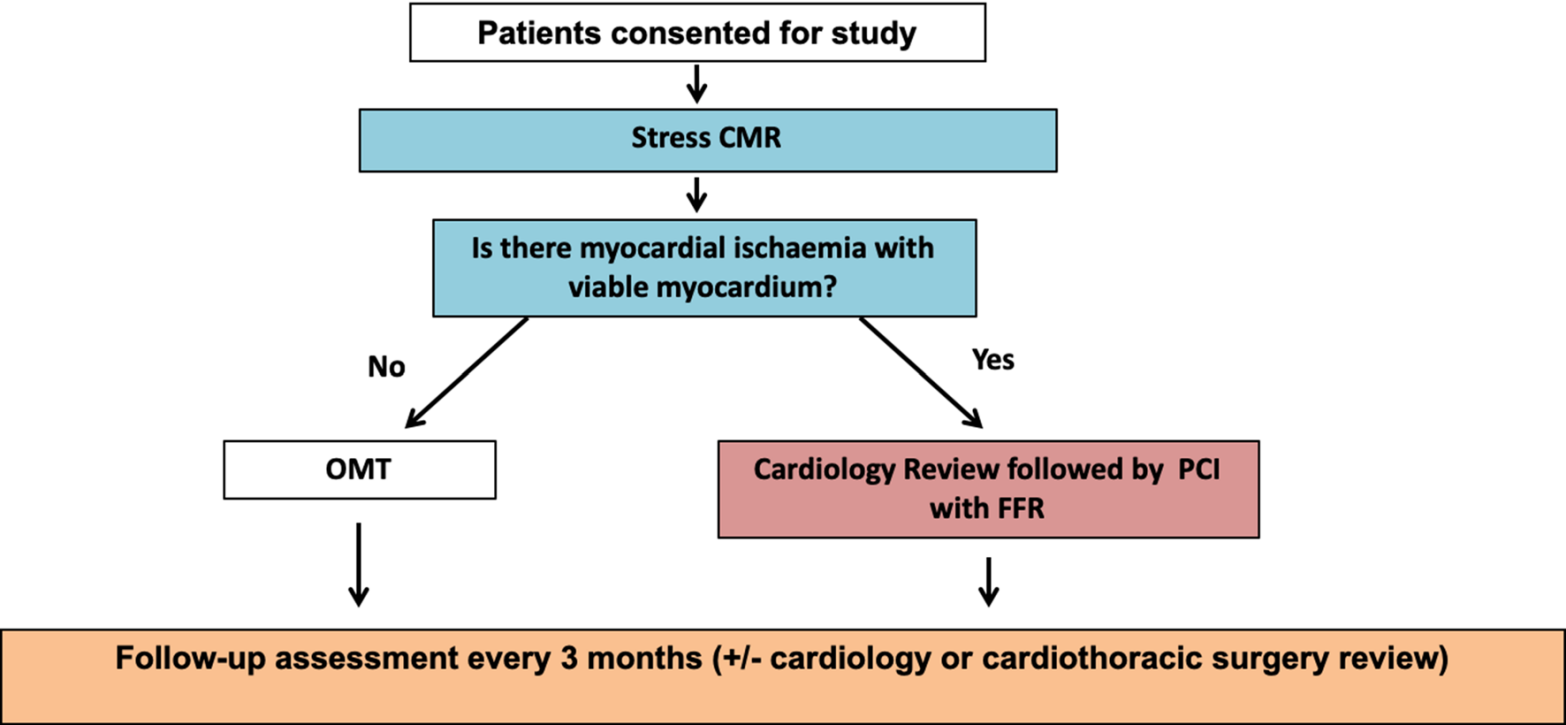
Study design. *CMR* cardiac magnetic resonance, *OMT* optimised medical therapy, *FFR* fractional flow reserve

All recruited patients underwent stress CMR examinations (Fig. 2).

**Fig. 3:**
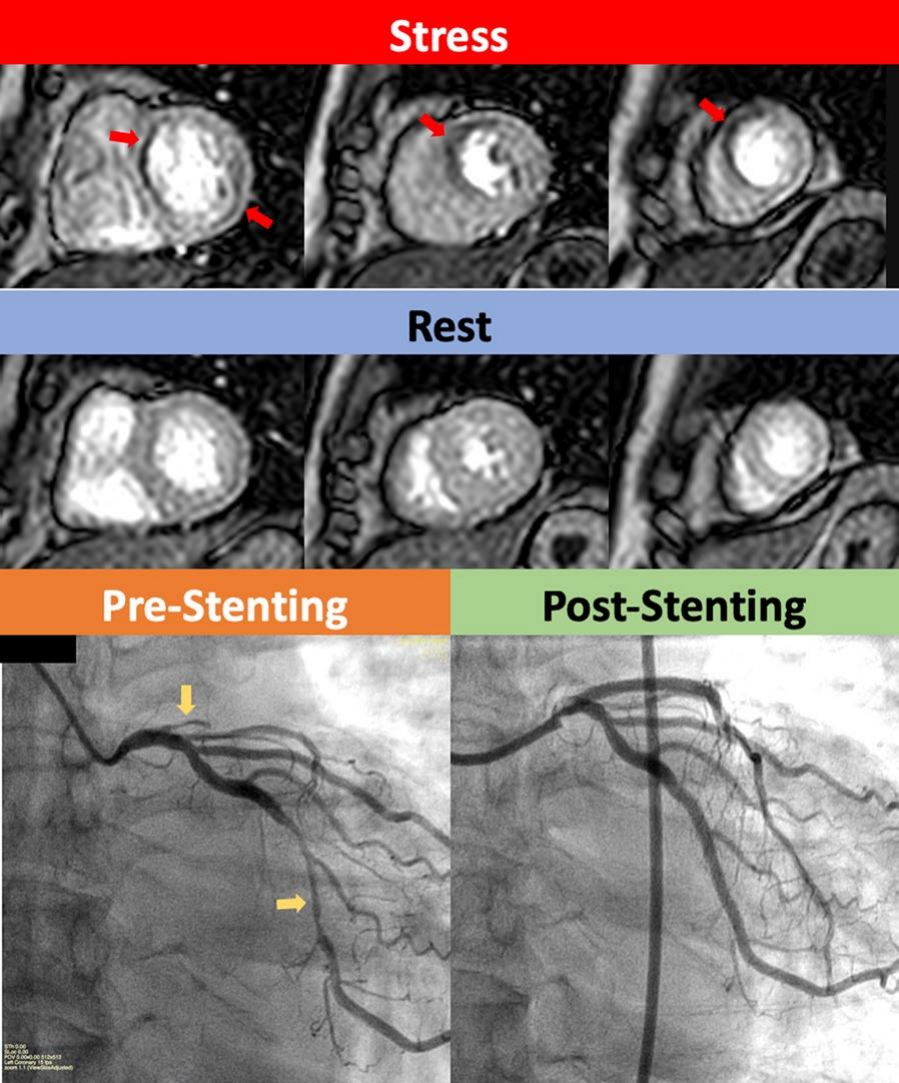
Asymptomatic male patient with type 2 diabetes mellitus who was actively hiking. This patient was recruited into our study and had a positive screening stress cardiac magnetic resonance. Top row of images show stress perfusion defects (red arrows) in the basal, mid-ventricular and apical slices in the left anterior descending (LAD) territory as well as a stress perfusion defect in the left circumflex (LCx) territory on the basal slice in the inferolateral wall (red arrow). These perfusion defects resolved on the rest images. The catheter coronary angiogram showed chronic total occlusion of the LAD (yellow arrow) and obstructive CAD of the LCx (yellow arrow). Fractional flow reserve measurements of the LCx was 0.69. Post-stenting image in the bottom right shows the re-perfusion of the LAD and expansion of the LCx narrowing

Patients with positive stress CMR examinations (see below for definition of positive/negative study) were referred to cardiology clinic to arrange catheter coronary angiography (CCA) with or without FFR (see Fig. 3). At the time of catheterisation if deemed appropriate by the cardiologist, 2nd generation drug eluting coronary stents were inserted if the FFR is ≤ 0.8 or coronary artery narrowing was ≥ 70%.

Patients with a negative stress CMR examination would return to family practice or diabetes clinics to have optimised medical therapy and follow-up clinic appointments.

### CMR protocol

All acquisitions were performed on a 3T Philips Achieva TX scanner, Philips Best, The Netherlands) and patients underwent multiplanar cine balanced steady state free precession imaging, stress/rest perfusion imaging and late gadolinium enhancement imaging.

### Stress and rest perfusion CMR technique

Three short axis stress and rest perfusion images were acquired in the left ventricular (LV) basal, mid and apical aspects. For the stress and rest images, a T1 weighted fast gradient echo sequence was utilised [slice thickness 10 mm, echo time (TE) 1.2 ms, repetition time (TR) 2.5 ms, flip angle 20°, field of view 320 mm × 320 mm].

Intravenous adenosine was administered (0.14 mg/kg/min) for up to 5 min. If inadequate stress was achieved, infusion rate was increased by 50% as previously described [9]. First pass stress perfusion was acquired at peak stress with intravenous injection of 0.05 mmol/kg of gadoterate meglumine (injection rate: 3 to 4 ml/s, with a subsequent 30 ml saline flush at the same flow rate). After discontinuation of intravenous adenosine and a 10 min resting period allowing for sufficient contrast agent elimination, resting first pass perfusion imaging was performed with an additional injection of 0.05 mmol/kg of gadoterate meglumine.

An additional 0.1 mmol/kg was given prior to acquiring the late gadolinium enhancement (LGE) images after 8–15 min after the second gadoterate meglumine injection for rest perfusion images.

### Definition of positive and negative stress CMR examinations

A positive study was defined as a study demonstrating a stress-induced perfusion defect. A stress-induced perfusion defect was defined as a dark sub-endocardial rim which lasts > 6 heart beats on the stress images, larger than 1 pixel breadth and was not present on the rest images. A negative study is a study without a stress induced perfusion defect.

A positive stress CMR study was regarded as indicative of myocardial ischaemia that would likely benefit from coronary artery stenting. A negative stress CMR was considered a study which did not demonstrate evidence of myocardial ischaemia and thus would not benefit from coronary artery stenting.

### Data post-processing and interpretation

CMR42 (Circle Inc., Calgary, Canada) was utilised to assess LV volumes, LV mass and LV ejection fraction. LV contouring of the endocardial and epicardial surfaces was performed on the short axis cine images in end-diastole and end-systole. Volumes and mass were corrected for body surface area. Body surface area was calculated using the Mosteller equation [10]. Calculation of the global MPRI was performed using semi-automated analysis (CMR42, Circle CVI, Calgary, Canada) by contouring the stress and rest perfusion images as previously described [6].

One blinded CMR analyst (WZ) performed the global MPRI measurements and the intraobserver measurements on 20 randomly selected subjects > 6 months apart. A second CMR analyst (BC) performed the interobserver measurements.

Briefly, the blood pool and LV endocardial and epicardial borders were contoured on each stress and rest perfusion image to determine the stress/rest signal intensity up-slope ratios and normalised for the arterial input function. For patients without obstructive CAD and global MPRI was < 1.4, this was classified as microvascular disease [6].

Images were assessed and reported for ischaemia, infarct and cardiac function by two fellowship trained cardiac radiologists through consensus (MYN, VV) with more than 10 years experience. If consensus could not be reached, an independent 3rd cardiac radiologist (BJW) would review the case.

### Catheter coronary angiography and FFR

CCA and FFR was performed by two interventional cardiologists. FFR was undertaken if it could be done safely and there was luminal narrowing of 40–90%. If FFR could not be performed, degree of coronary artery narrowing was determined by two interventional cardiologists at the time of procedure. At the time of catheterisation, 2^nd^ generation drug eluting coronary stents were inserted if FFR ≤ 0.8 or coronary artery narrowing was ≥ 70%.

### Fractional flow reserve procedure

FFR was performed using a pressure wire at maximal hyperaemia. Hyperaemia was induced via infusion of 0.14 mg/kg/min of adenosine intravenously. FFR was calculated as the mean distal coronary artery pressure divided by the mean aortic pressure during maximal hyperaemia.

### Patient follow-up

Patients in both groups were followed up to ensure optimization of medical treatment and that CCA were performed in a timely manner. Major adverse cardiovascular events (MACE) were recorded (i.e. Death, heart failure, hospitalisation for acute coronary syndrome, stroke). This was done via out-patient clinics, review of the national patient electronic record system and telephone contact every three months. All patients were successfully followed up.

### Statistical analysis

Primary outcome measures were (1) myocardial ischaemia detected by CMR and confirmed on CCA, (2) global MPRI of asymptomatic DM patients compared to normal volunteers. Myocardial ischaemia prevalence is presented as percentages. With the small number of volunteers, non-parametric tests were utilised (i.e. Mann–Whitney U tests and Fisher’s exact test) to compare asymptomatic DM patients and healthy volunteers. Spearman’s correlation was performed to correlate global MPRI with various continuous variables. To determine inter and intraobserver variability for global MPRI measurements, bias and limits of agreement were determined in 20 randomly selected cases. McNemar’s Chi squared test was used to compare medication prescriptions before and 1 year after stress CMR examination. A p value < 0.05 was considered statistically significant.

## Results

63 patients were recruited. Our patient cohort were predominantly Chinese males (77.8%) and high risk based on the Framingham Risk score (mean 36.4%, SD 13.2%). Our healthy volunteer cohort (n = 7) was slightly younger in age but not significantly different for gender. Detailed patient and volunteer characteristics are presented in Table 1.

**Table 1:** Patient and normal volunteer cohort characteristics (n = 70)

Characteristic	Asymptomatic patients with DM (n = 63)	Healthy volunteers (n = 7)	p
Age (years)	66.3±4.4	60.4±4.1	0.005*
Male	77.8%	42.8%	0.07
Body mass index (kg/m^2^)	25.8±3.9	21.1±2.6	0.003*
DM duration (years)	15.8±8.8	–	–
HbA1c (%)	7.2±1.0	–	–
Cardiovascular risk factors			
Systolic blood pressure (mmHg)	138.1±9.0	133.3±5.3	0.14
Smoking	11.1%	0%	0.46
High density lipoprotein (mmol/L)	1.30±0.46	1.53±0.43	0.10
Total cholesterol (mmol/L)	4.10±0.71	5.26±0.40	< 0.001*
Diabetic complications			
eGFR (ml/min/1.73 m^2^)	76.6±18.6	87.9±10.6	0.04
Microalbumin/creatnine ratio (mg/mmol)	7.89±9.11	–	–
Retinopathy	14%	–	–
Peripheral vascular disease	1.4%	–	–
Anti-hypertensives			
Beta-blockers	17.5%	0%	0.59
ACE inhibitor	46.0%	0%	0.04
Calcium channel blockers	65.1%	0%	< 0.01*
Alpha-blockers	7.9%	0%	1.00
Diuretics	7.9%	0%	1.00
Angiotensin receptor blocker	19.0%	0%	0.34
Lipid-modifying agents			
Statin	61.9%	0%	< 0.01*
Fibrates	1.6%	0%	1.00
Anti-hyperglycaemic agents			
Acarbose	1.6%	0%	1.00
Sulfonylureas	36.5%	0%	0.09
SGLT2 inhibitor	17.5%	0%	0.59
Metformin	90.5%	0%	< 0.01*
DDP-4 inhibitor	41.3%	0%	0.04
Insulin	30.2%	0%	0.18
Pioglitazone	22.2%	0%	0.33
Anti-platelet agents			
Clopidogrel	6.3%	0%	1.00
Aspirin	23.8%	0%	0.33

*DM* diabetes mellitus type 2, *HbA1C* glycated haemoglobin, *eGFR* estimated glomerular filtration rate, *ACE* angiotension converting enzyme, *SGLT*-2 sodium-glucose co-transport 2 inhibitor, *DDP*-4 dipeptidyl peptidase-4

*p < 0.05

LV and left atrial parameters are stated in Table 2. Compared to normal volunteers, patients had significantly higher LV mass index (p = 0.003) and smaller left atrial area index (p = 0.04).

**Table 2:** Patient cohort’s CMR parameters

CMR parameters	Asymptomatic DM patients (n = 63)	Healthy volunteers (n = 7)	p
LVEDV indexed (ml/m^2^)	75.4 ± 14.8	83.9 ± 18.8	0.19
LVESV indexed (ml/m^2^)	32.3 ± 10.5	43.3 ± 26.8	0.12
Stroke volume (ml)	75.7 ± 13.6	76.0 ± 25.9	0.88
LVEF (%)	57.6 ± 6.3	56.6 ± 6.6	0.39
LV mass indexed (g/m^2^)	42.8 ± 11.3	28.5 ± 8.2	0.003*
Cardiac output (L/min)	5.50 ± 1.10	4.83 ± 1.63	0.26
Left atrial area indexed (cm^2^/m^2^)	13.2 ± 2.4	15.0±1.9	0.04*
Right atrial area indexed (cm^2^/m^2^)	11.7 ± 2.2	13.3 ± 2.5	0.15
LGE infarcts	5 (7.9%)	0 (0%)	1.00
Global MPRI	1.42 ± 0.28	1.83 ± 0.31	0.003*

Indexed parameters were corrected using body surface area

*CMR* cardiac magnetic resonance, *DM* diabetes mellitus type 2, *LVEDV* left ventricular end-diastolic volume, *LVESV* left ventricular end-systolic volume, *LVEF* left ventricular ejection fraction, *LV* left ventricle, *MPRI* myocardial perfusion reserve index

*p < 0.05

25 patients had positive stress CMR examinations. 5 patients (7.9%) had infarcts detected of which only 2 of these patients with infarcts had no evidence of stress induced perfusion defects.

One patient’s images had very poor image quality and could not be analysed for global MPRI. This patient was excluded from the analysis. Asymptomatic DM patients had lower global MPRI than normal volunteers (1.43 ± 0.27 vs 1.83 ± 0.31 respectively; p < 0.01). After excluding patients with obstructive CAD (n = 50), global MPRI remained significantly lower in patients (1.45 ± 0.27 vs 1.83 ± 0.31 respectively; p < 0.01). Amongst 49 patients, 51.0% (n = 25) had global MPRI < 1.4 and thus could be classified as having microvascular disease.

Global MPRI correlated with age (r = − 0.28, p = 0.021), BMI (r = − 0.31, p = 0.009) and estimated glomerular filtration rate (r = 0.26, p = 0.031). Other biochemical and CMR parameters did not show any significant correlation (see Table 3). For intraobserver variability, the bias was 0.015 and levels of agreement − 0.12 to 0.15. For interobserver variability, the bias was 0.061 and levels of agreement − 0.16 to 0.29.

**Table 3:** Spearman’s correlation coefficients between myocardial perfusion reserve index (MPRI) and clinical parameters, biochemical and cardiac magnetic resonance parameters in patients and normal volunteers (n = 70)

Characteristic	MPRI	
	Coefficient	p
Age (years)	− 0.28	0.021*
BMI (m^2^)	− 0.31	0.009*
Diabetes duration (years)	0.00	1.00
HbA1c (%)	0.22	0.08
Systolic blood pressure (mmHg)	− 0.19	0.12
HDL (mmol/L)	0.13	0.29
Total cholesterol (mmol/L)	0.17	0.15
eGFR (ml/min/1.73 m^2^)	0.26	0.031*
Microalbumin/creatnine ratio (mg/mmol)	0.01	0.96
Framingham risk score (%)	− 0.23	0.06
CMR parameters		
LVEDV indexed (ml/m^2^)	0.18	0.15
LVESV indexed (ml/m^2^)	0.10	0.41
LVEF (%)	0.07	0.56
LV mass indexed (g/m^2^)	− 0.05	0.68
Left atrial area indexed (cm^2^)	0.05	0.70

*BM* body mass index, *HbA1C* glycated haemoglobin, *HDL* high density lipoprotein, *eGFR* estimated glomerular filtration rate, *LVEDV* left ventricular end-diastolic volume, *LVEF* left ventricular ejection fraction, *LV* left ventricular

*p < 0.05

22 of 25 patients with positive stress CMR (88%) agreed to undergo CCA. 3 patients declined to proceed with CCA.

Of the 22 patients undergoing CCA, 31 vessels (47%) had FFR performed. In total, 13 patients (20.6%) had positive stress CMR examinations with confirmed obstructive CAD on CCA. Of these 13 patients, 9 patients had FFR ≤ 0.8 and a further 4 patients had coronary artery narrowing ≥ 70%. 2 of the 9 patients with FFR < 0.8 had complete occlusion of a coronary artery whilst 1 of the 4 patients with coronary artery narrowing ≥ 70% had complete occlusion of a coronary artery. FFR could not be measured in these 3 occluded vessels. On a per vessel analysis, the number of correctly identified ischaemic territories (i.e. true positives) were 11 for left anterior descending artery, 3 for left circumflex artery and 4 for right coronary artery. See Table 4 for the per vessel analysis of all three coronary arteries.

**Table 4:** Per vessel analysis of positive stress CMR results based on the 22 patients that underwent coronary catherization

	CMR LAD territory +ve	CMR LAD territory −ve
LAD		
Cath LAD territory +ve	11	2
Cath LAD territory –ve	7	2
LCx		
Cath LCx territory +ve	3	1
Cath LCx territory –ve	6	12
RCA		
Cath RCA territory +ve	4	1
Cath RCA Territory –ve	6	11

In total, 12 patients had coronary artery stents inserted, with 1 patient declining coronary artery stent placement. Of the 12 patients, 3 patients had 2 stents inserted whilst 9 patients had 1 stent inserted.

Patient follow-up 1 year post stress CMR demonstrated significant changes in medication prescribed to patients as compared to pre CMR status (see Table 5). In the whole cohort, there was an increase in beta-blocker (p = 0.014), statin (p = 0.0067), sodium-glucose co-transport 2 inhibitors (SGLT-2) (p = 0.025), thiazolidinediones (p = 0.025), clopidogrel (p = 0.0047) and aspirin prescription (p = 0.0013). When patients were divided into those with normal and abnormal stress CMR (see Table 6), calcium channel blockers (p = 0.03), statins ((p = 0.01), clopidogrel (p = 0.001) and aspirin (p < 0.001) prescription was significantly increased in patients with abnormal stress CMR. Over a median of 653 days (range 422–780 days), there was no death, heart failure, hospitalisation for acute coronary syndrome or stroke.

**Table 5:** Cardiovascular and diabetic drug therapy from scan to 1 year post scan

	Baseline	1 year post scan	p
Anti-hypertensives			
Beta-blockers	11 (17%)	17 (27%)	0.014*
ACE inhibitor	29 (46%)	33 (52%)	0.10
Calcium channel blockers	41 (65%)	44 (69%)	0.18
Alpha-blockers	5 (8%)	7 (11%)	0.32
Diuretics	5 (8%)	8 (13%)	0.08
ARB	12 (19%)	13 (21%)	0.32
Lipid-modifying agents			
Statin	39 (62%)	48 (76%)	0.0067*
Fibrates	1 (2%)	1 (2%)	1
Anti-hyperglycaemic agents			
Acarbose	1 (2%)	1 (2%)	1
Sulfonylureas	23 (37%)	23 (37%)	1
SGLT-2 inhibitor	11 (17%)	16 (25%)	0.025*
Metformin	57 (90%)	59 (94%)	0.16
DDP-4 inhibitor	26 (41%)	27 (43%)	0.71
Insulin	19 (30%)	19 (30%)	1
Pioglitazone	14 (22%)	19 (30%)	0.025*
Anti-platelet agents			
Clopidogrel	4 (6%)	12 (19%)	0.0047*
Aspirin	15 (24%)	27 (43%)	0.0013*

*ACE* angiotension converting enzyme, *ARB* angiotensin receptor blocker, *SGLT*-2 sodium-glucose co-transport 2 inhibitor, *DDP*-4 dipeptidyl peptidase-4

* p < 0.05

**Table 6:** Change in cardiovascular and diabetic drug therapy 1 year post scan based on having a normal or abnormal stress perfusion cardiac magnetic resonance (CMR) examination

	1 year change in medications		
	Normal CMR (n = 36)	Abnormal CMR (n = 27)	p
Anti-hypertensives			
Beta-blockers	+2	+4	0.39
ACE inhibitor	+1	+3	0.80
Calcium channel blockers	−1	+4	0.03*
Alpha-blockers	0	+2	0.76
Diuretics	0	+3	0.07
ARB	+1	0	1.00
Lipid-modifying agents			
Statin	+1	+8	0.01*
Fibrates	0	0	1.00
Anti-hyperglycaemic agents			
Acarbose	−1	+1	0.68
Sulfonylureas	0	0	1.00
SGLT-2 inhibitor	+2	+3	0.64
Metformin	+1	+1	1.00
DDP-4 inhibitor	+2	−1	0.23
Insulin	0	+1	0.68
Pioglitazone	+3	+2	1.00
Anti-platelet agents			
Clopidogrel	0	+8	0.001*
Aspirin	0	+12	< 0.001*

Abnormal stress CMR was defined as having a stress perfusion defect and/or infarct

*ACE* angiotension converting enzyme, *ARB* angiotensin receptor blocker, *SGLT*-2 sodium-glucose co-transport 2 inhibitor, *DDP*-4 dipeptidyl peptidase-4

*p < 0.05

## Discussion

To the best of our knowledge, this is the first study to investigate a stress perfusion CMR screening programme for high-risk asymptomatic DM patients. There were two main findings: (1) 20.6% of patients had a positive stress CMR examination with obstructive CAD and (2) asymptomatic DM patients had lower global MPRI compared to normal volunteers.

### Prevalence of silent obstructive CAD & silent infarcts

Our 20.6% prevalence of myocardial ischaemia in asymptomatic DM patients is in keeping with previously published results of 17–59% which utilised nuclear myocardial perfusion and exercise stress testing [1, 11, 12]. This data can now be used for more accurate sample size calculations if larger screening trials are planned. Furthermore, it should be noted that the published prevalence of silent ischaemia is based predominantly on imaging findings without catheter coronary angiography confirmation, thus we believe our stress CMR findings confirmed by angiography provide a more robust estimate of the prevalence of silent obstructive CAD in DM patients rather than just ischaemia detected on non-invasive imaging [12].

In our cohort, 7.9% of patients had silent myocardial infarcts which was consistent with previous studies (i.e. 1.9–17.5% of silent myocardial infarcts in asymptomatic DM patients) [13–16]. How these findings alters patient management and whether this alters patient outcome has not been directly studied. This study though provides some insight by demonstrating increases in statin, clopidogrel and aspirin prescriptions as a result of an abnormal stress CMR examination. Furthermore, we found no major adverse cardiovascular events occurred in this cohort, but further study is warranted to assess the effect of stress CMR screening in terms of patient outcome.

### Coronary intervention based on stress perfusion CMR findings

The insertion of coronary artery stents in asymptomatic patients with type 2 diabetes and obstructive CAD is not recommended in guidelines [17, 18]. However, silent ischaemia in patients with type 2 diabetes is well established but the management of silent ischaemia in light of abnormal functional imaging findings is less certain. Indeed, the European Society of Cardiology has suggested that functional imaging could be utilised for silent ischaemia screening but the management of abnormal findings still requires further evidence [17]. At present both, European and American guidelines lack studies utilising stress perfusion CMR for ischaemia detection and prediction of inteventional outcomes in presence of stress perfusion CMR identified ischemia. Therefore this study provides the first patient outcomes as a result of screening with stress CMR and active intervention with coronary stenting. Furthermore, studies have frequently grouped stress perfusion CMR with other functional imaging studies [19] but various randomised controlled studies and meta-analyses have shown that stress perfusion CMR has superior diagnostic accuracy to many other functional studies as well as being non-inferior to FFR [5, 20–22]. Therefore, further research using stress perfusion CMR is warranted to determine if it may have a role in screening asymptomatic patients with type 2 diabetes.

### Global MPRI and MCAD—potential therapeutic target

Using global MPRI, we demonstrated that asymptomatic DM patients had lower myocardial perfusion than normal volunteers. Moreover, > 50% of patients have evidence of MCAD. Previous studies have demonstrated reduced myocardial perfusion in patients with type 2 diabetes with chest pain [23], utilising only rest perfusion technique [24] and asymptomatic patients without investigating for obstructive coronary artery disease [25]. However, our study differs in that it establishes the proportion of patients with obstructive coronary artery disease, MCAD and those without microvascular disease for potential targeted therapy.

MCAD is a well established complication in type 1 and 2 diabetes mellitus [26]. MCAD carries significant independent prognostic significance as Murthy et al. previously demonstrated [7]. In their study, MCAD in DM patients was an independent predictor of MACE, and the risk was comparable to non-DM patients with obstructive CAD. MCAD has therefore increasingly become a focus of research interest to help reduce MACE in DM patients. However, there is currently no proven therapy to treat the condition.

Nonetheless, our sample size is small so further assessing global MPRI to identify microvascular dysfunction in diabetic patients and its prognostic significance needs to be undertaken. However, global MPRI or full quantification of myocardial blood flow on stress CMR [27] could represent a promising biomarker for targeting higher risk DM patients rather than identifying only obstructive CAD via the current clinical practice of identifying stress induced perfusion defects. This study adds to the growing literature of potential magnetic resonance imaging markers such as aortic stiffness [28], abdominal adiposity [29] and high signal coronary artery plaque characterisation [30] in patients with type 2 diabetes that have shown promise in identifying patients with increased cardiovascular risk or adverse cardiac remodelling.

## Limitations

Firstly, this was a small observational study to demonstrate the feasibility of utilising stress CMR in screening asymptomatic DM patients. Nonetheless, our study included outcome data which will allow sample size calculations to be performed for larger screening trials. Secondly, FFR was not performed in all coronary arteries. However, this likely represents real world situations where due to multiple factors FFR is not performed on all coronary arteries [31] such as narrowings < 50% or if the cardiologist deems there is a significant risk to patients if FFR is undertaken.

Thirdly, CCA was not performed in all patients so the true burden of obstructive CAD cannot be known. However, it would be hard to justify ethically to perform CCA on asymptomatic patients with type 2 diabetes with no evidence of ischaemia on stress CMR.

Lastly, our study did not set out to answer or refine the DM patients which would benefit most from screening. Using the Framingham risk score would lead to preferential recruitment of males as our study demonstrates. If MCAD or global MPRI is to be utilised as a marker for medical intervention, an alternative risk predictor may need to be utilised since women more commonly have MCAD and non-obstructive coronary arteries compared to males [32, 33]. Other screening trials have previously set ≥ 2 risk factors as an inclusion criteria for DM patients [34, 35]. This can prevent a recruitment bias towards males but further studies are required to determine the ideal cohort. Furthermore, there are a myriad of DM cardiovascular risk calculators but these best predict risk in cohorts with similar geographical location and ethnicity from which the studies originate [36–38]. This means that risk calculators may not be generalisable to other populations. Nonetheless, we chose the Framingham risk calculator as it is a widely accepted and validated risk calculator, even though its accuracy may be lower in certain ethnic groups.

## Conclusion

This feasibility study on stress CMR screening of high risk asymptomatic DM patients demonstrated a 20.6% prevalence of myocardial ischaemia as confirmed by CCA with or without FFR and lower global MPRI in DM patients than normal volunteers.

## Data Availability

The datasets used and/or analysed during the current study are available from the corresponding author on reasonable request.

## Abbreviations

CAD: Coronary artery disease
CCA: Catheter coronary angiography
CMR: Cardiovascular magnetic resonance
DM: Diabetes Mellitus type 2
FFR: Fractional flow reserve
LV: Left ventricle
MACE: Major adverse cardiovascular events
MCAD: Microvascular coronary artery disease
MPRI: Myocardial perfusion reserve index
SGLT-2: Sodium-glucose co-transport 2 inhibitors
